# An arrayed CRISPR knockout screen identifies genetic regulators of GLUT1 expression

**DOI:** 10.1038/s41598-023-48361-5

**Published:** 2023-11-29

**Authors:** Yajuan Shi, Ketaki A. Katdare, Hyosung Kim, Jonah C. Rosch, Emma H. Neal, Sidney Vafaie-Partin, Joshua A. Bauer, Ethan S. Lippmann

**Affiliations:** 1 Department of Chemical and Biomolecular Engineering, Vanderbilt University, Nashville, TN USA; 2 Vanderbilt Brain Institute, Vanderbilt University, Nashville, TN USA; 3 Vanderbilt Institute of Chemical Biology, Vanderbilt University, Nashville, TN USA; 4 Department of Biochemistry, Vanderbilt University, Nashville, TN USA; 5 Department of Biomedical Engineering, Vanderbilt University, Nashville, TN USA; 6 Vanderbilt Center for Stem Cell Biology, Vanderbilt University, Nashville, TN USA; 7 Interdisciplinary Materials Science Program, Vanderbilt University, Nashville, TN USA; 8 Department of Neurology, Vanderbilt University Medical Center, Nashville, TN USA; 9 Vanderbilt Memory and Alzheimer’s Center, Vanderbilt University Medical Center, Nashville, TN USA

**Keywords:** High-throughput screening, Cell signalling

## Abstract

Glucose, a primary fuel source under homeostatic conditions, is transported into cells by membrane transporters such as glucose transporter 1 (GLUT1). Due to its essential role in maintaining energy homeostasis, dysregulation of GLUT1 expression and function can adversely affect many physiological processes in the body. This has implications in a wide range of disorders such as Alzheimer’s disease (AD) and several types of cancers. However, the regulatory pathways that govern GLUT1 expression, which may be altered in these diseases, are poorly characterized. To gain insight into GLUT1 regulation, we performed an arrayed CRISPR knockout screen using Caco-2 cells as a model cell line. Using an automated high content immunostaining approach to quantify GLUT1 expression, we identified more than 300 genes whose removal led to GLUT1 downregulation. Many of these genes were enriched along signaling pathways associated with G-protein coupled receptors, particularly the rhodopsin-like family. Secondary hit validation confirmed that removal of select genes, or modulation of the activity of a corresponding protein, yielded changes in GLUT1 expression. Overall, this work provides a resource and framework for understanding GLUT1 regulation in health and disease.

## Introduction

Glucose is a key energy source for the human body. Circulating blood carries glucose to all organs of the body where it is taken up by cells with the help of specialized membrane transporters. The uptake of glucose by cells is facilitated by the GLUT (SLC2) family of membrane transporters^1^. Of these, GLUT1 has been extensively studied with a well characterized gene sequence and crystal structure^2–4^. It is one of the key transporters responsible for regulating cellular glucose uptake, thus maintaining metabolic homeostasis^5, 6^. Although ubiquitously expressed, particularly high levels of GLUT1 are found in erythrocytes and blood–brain barrier (BBB) endothelial cells, which provides a continuous supply of glucose to meet high energy demands^7, 8^. Interestingly, in addition to its role in glucose homeostasis, GLUT1 can respond to hypoxic stress and provide protection against mitochondrial oxidative injury^9–11^. This is in part due to its ability to also facilitate transport of ascorbic acid^12^.

GLUT1 is essential for the normal functioning of various physiological processes such as embryo development, transplacental movement of glucose, angiogenesis in the central nervous system (CNS), and T cell activation^13–16^. Deficiency in expression and function of GLUT1 is associated with GLUT1 deficiency syndrome, a complex disorder characterized by altered brain homeostasis resulting in developmental delays, epilepsy, and movement disorders^17–19^. Additionally, glucose hypometabolism, which is associated with reductions in cerebrovascular GLUT1 expression, can predict cognitive decline in normal aging subjects and progression of Alzheimer’s disease (AD)^20–30^. Further, elevated GLUT1 expression has been observed in many types of cancers and has been the subject of scrutiny for potential therapeutic benefits^31–34^. Hence, therapies aimed at altering GLUT1 transporter expression could be beneficial for many patients^35^. However, regulation of GLUT1 remains a poorly understood process, which prevents rational attempts to overcome loss of transporter expression.

Identifying genetic factors that contribute to the downregulation of GLUT1 expression is key to understanding the pathophysiological mechanisms that govern conditions associated with impaired GLUT1 function. To better understand the mechanisms underlying GLUT1 regulation, we performed an arrayed CRISPR knockout screen in Caco-2 epithelial cells. Using this cell line, we screened more than 8,000 genes and measured GLUT1 expression on a per cell basis using automated high content immunostaining. From this approach, we identified more than 300 genes whose removal reduced GLUT1 expression. Pathway analyses revealed enrichment along G-protein coupled receptor (GPCR) and purinergic signaling. We validated several genes and pathways of interest via gene knockout or pharmacological manipulation. Overall, we expect this study will provide important insights into GLUT1 regulation and help develop strategies to restore GLUT1 expression for therapeutic benefit. Moreover, the framework from this study may prove useful in generally guiding image-based CRISPR screens focused on membrane transporters.

## Methods

### Caco-2 cell medium and matrix formulations

Complete growth medium was prepared with DMEM (Corning #10-013-CV) supplemented with 10% heat-inactivated fetal bovine serum (FBS, Thermo Fisher Scientific #26140079), and 1X MEM Non-essential amino acid solution (Sigma-Aldrich #M7145). For heat inactivation, FBS was thawed completely at room temperature, heated at 56 °C in the water bath for 30 min, and cooled on ice immediately.

Lentiviral transduction medium was prepared with 495 mL of DMEM and 5 mL of MEM Non-essential amino acid solution.

Single cell sorting medium was prepared with phenol-red free DMEM (Fisher Scientific #21-063-029) supplemented with 10 μM Y27632 (Tocris #1254) and 1× penicillin–streptomycin (Thermo Fisher Scientific #15140122).

Bulk cell sorting medium was prepared with phenol-red free DMEM supplemented with 10 μM Y27632 and 1× Antibiotic–Antimycotic (100×, Thermo Fisher Scientific #15240062).

2× anti-anti medium was prepared with 490 mL of complete growth medium supplemented with 10 mL of 1× Antibiotic–Antimycotic.

1× anti-anti medium was prepared with 495 mL of complete growth medium supplemented with 5 mL of Antibiotic–Antimycotic.

0.1% gelatin solution was prepared by solving 500 mg of gelatin (Sigma-Aldrich #G1890) into 500 mL of UltraPure DNase/RNase-free distilled water (Thermo Fisher Scientific #10977015) at 55 °C.

### Caco-2 cell maintenance

Caco-2 cells were maintained in complete growth medium on 0.1% gelatin-coated 6-well plates (Fisher Scientific #07-200-83) at 37 °C in a humidified CO_2_ incubator. Medium was changed every 2 days. Cells were passaged every 6 days upon reaching 80–100% confluency. For passaging, cells were washed once with DPBS (Fisher Scientific #14-190-144), dissociated with TrypLE Express (Fisher Scientific #12604013) for 5–7 min, collected via centrifugation, and resuspended into complete growth medium. For screening experiments, cells were maintained in T175 tissue culture-treated flasks (Corning #431080). Cells were cultured in complete growth medium unless otherwise noted.

### Generation of a clonal Cas9-expressing Caco-2 line

Caco-2 cells were seeded onto 2 wells of a 24-well cell culture plate (Fisher Scientific #09-761-146) coated with 0.1% gelatin at the density of 6 × 10^4^ cells/well. 24 h after seeding, a well of cells was used to measure density with the Countess II automated cell counter (Thermo Fisher Scientific). Cells in the other well were transduced with lentiviral particles encoding *S. pyogenes* Cas9 (Dharmacon #VCAS11865) at the multiplicity of infection of 0.3. Lentiviral particles were delivered in 0.25 mL of lentiviral transduction medium supplemented with 5 μg/mL polybrene (Sigma-Aldrich #TR-1003-G). The delivered transgene is driven by the constitutive hEF1ɑ promoter and also contains the mKate2 fluorescent reporter. 5 h after the initiation of transduction, 0.75 mL of complete growth medium was added to the cells. 48 h later, cells were fully switched to complete growth medium and expanded for fluorescence-activated cell sorting (FACS).

To generate a monoclonal Cas9-expressing line, cells were washed with DPBS, dissociated with TrypLE Express, collected via centrifugation, and resuspended into single cell sorting medium at the density of 5 × 10^6^ cells/mL. Single cells were sorted based on the expression level of mKate2 using a 5-laser FACS Aria III (BD Biosciences) with a 100-μm nozzle. Sorted clones were plated onto a gelatin-coated 96-well cell culture plate containing 100 μL of single cell sorting medium supplemented with 10 mM HEPES (Thermo Fisher Scientific #15630080). 24 h after sorting, 100 μL of complete growth medium was added to each well. 48 h after sorting, medium was fully switched to complete growth medium. Several clones were screened for Cas9 expression, and one denoted as C6 (C6-Caco-2 cells) was selected for use moving forward.

### Generation of C6-Caco-2 cells with homogeneous GLUT1 expression

Our prior work has shown that GLUT1 is heterogeneously expressed in Caco-2 cells^36^. To create a C6-Caco-2 cell line with more uniform GLUT1 expression, we performed bulk FACS. C6-Caco-2 cells were cultured on 0.1% gelatin-coated plates until they reached 80% confluency. Cells were washed with DPBS, dissociated with TrypLE Express, and collected via centrifugation. Collected pellets were resuspended into bulk cell sorting medium supplemented with Alexa Fluor 488 primary conjugated GLUT1 antibody (Abcam #FAB1418G, 1:1000 dilution) at the density of 5 × 10^6^ cells/mL. Approximately 2% of cells with highest fluorescence intensity were sorted into one well of 0.1% gelatin-coated 12-well plate using a 5-laser FACS Aria III with a 100-μm nozzle (Supplementary Fig. 1). 24 h after sorting, 1 mL of 2× anti-anti medium was added to each well. 48 h after sorting, cells were fully switched to 1× anti-anti medium and then expanded normally. This cell line is referred to as GLUT1^high^-C6-Caco-2.

### Western blot

Cells were rinsed once with DPBS, scraped with the cell lifter (Fisher Scientific #08-100-240), and collected via centrifugation. Cells were lysed in RIPA buffer (Sigma Aldrich #R0278) supplemented with 1% v/v of protease inhibitor cocktail (Sigma-Aldrich #P8340) and 1% v/v of phosphatase inhibitor cocktail 3 (Sigma-Aldrich #P0044). After a 30-min lysis on ice, samples were centrifugated at 12,000xg for 15 min at 4 °C and the supernatants were collected. Protein concentrations were measured with a Pierce BCA protein assay kit (Thermo Fisher Scientific #23225). Then, 25 μL of sample mixture was prepared containing Laemmli buffer (Bio-Rad #1610747), β-mercaptoethanol (Sigma-Aldrich #M3148), 25 μg of protein, and UltraPure DNase/RNase-free distilled water, and this mixture was run on a 4–20% Criterion TGX precast midi protein gel (Bio-Rad #5671094) and transferred onto a nitrocellulose membrane (Thermo Fisher Scientific #IB23001) using the iBlot 2 gel transfer device (Thermo Fisher Scientific #iB21001). After dry transfer, membranes were blocked with LiCor Odyssey blocking buffer (Li-Cor #92750000) at room temperature for 30 min, incubated with LiCor Odyssey blocking buffer supplemented with primary antibody and 0.05% Tween 20 (Millipore Sigma #P9416) at 4 °C overnight, and incubated with 1× Tris Buffered Saline (TBS, Corning #46-012-CM) supplemented with respective secondary antibody and 0.05% Tween 20 at room temperature for 2 h. Membranes were imaged with Odyssey XF imaging system (Li-Cor), and images were quantified with Image Studio. Primary and secondary antibodies are provided in Supplementary Tables 1 and 2.

### Immunocytochemistry

Fixed cells were blocked with DPBS containing 5% donkey serum (Millipore Sigma #D9663) and 0.3% Triton-X 100 (Sigma-Aldrich #X100), referred to as PBSDT, at room temperature for 1 h. Cells were then incubated with primary antibody diluted in PBSDT at 4 °C overnight. After primary antibody incubation, cells were washed three times with DPBS, incubated with secondary antibody diluted in PBSDT at room temperature for 2 h, incubated with 4′,6-diamidino-2-pheny-lindoldihydrochloride (DAPI, Thermo Fisher Scientific #D3571) diluted in DPBS for 10 min, and then washed with DPBS three times. Secondary labeling was skipped if primary antibody was conjugated with a fluorophore. Images were acquired using a Leica DMi8 microscope or an ImageXpress Micro XL Widefield high content screening system. Images collected on the DMi8 were analyzed with ImageJ and images collected on the ImageXpress were processed as described in the next section. Primary and secondary antibodies are provided in Supplementary Tables 1 and 2.

### Manual transfection strategy

Edit-R predesigned CRISPR RNA (crRNA, non-targeting or gene-targeting) and trans-activating RNA (tracrRNA, Dharmacon #U-002005) were resuspended in nuclease-free 10 mM Tris–HCl buffer pH 7.4 (Dharmacon #B-006000-100) at the desired final concentrations. Transfection mix was prepared in 20 μL of Opti-MEM I reduced serum medium (Thermo Fisher Scientific #319850-62) containing 0.5 μL of DharmaFECT1 reagent (Dharmacon #T-2001) and 125 nM of crRNA:tracrRNA complex. 20 min after incubation, 80 μL of GLUT1^high^-C6-Caco-2 cells in complete growth medium was seeded onto each well of the 0.1% gelatin-coated, flat-bottom, black-walled 96-well μCLEAR microplate (Greiner Bio-One #655096) at the density of 4.4 × 10^4^ cells/well. This resulted in each well containing 100 μL of complete growth medium supplemented with 25 nM of crRNA:tracrRNA and 0.5 μL of DharmaFECT1. 24 h after the initiation of transfection, 100 μL of 2× anti-anti medium was added to each well. 48 h after the initiation of transfection, medium was completely replaced with 200 μL of 1× anti-anti medium. 72 h after the initiation of transfection, cells were rinsed once with DPBS, fixed with 4% paraformaldehyde solution for 20 min (Thermo Fisher Scientific #AAJ19943K2), and washed once with DPBS. Plates were stored at 4 °C for downstream processing. A list of crRNAs is provided in Supplementary Table 3.

### Arrayed CRISPR knockout screen

Library plates were stamped prior to the screening. Libraries of human predesigned Edit-R crRNA in 96-well format were purchased from Dharmacon, covering genes across human transcription factors, protein kinases, GPCRs, proteases, ion channels, phosphatases, ubiquitin enzymes, and the druggable genome. Within each plate, a single well contained 0.1 nmol of four pooled crRNAs targeting the same gene. After brief centrifugation, crRNA was resuspended in 100 μL of nuclease-free 10 mM Tris–HCl pH 7.4 buffer using a Multidrop Combi Reagent Dispenser (Thermo Fisher Scientific #5840300) and incubated on an orbital shaker for 70 min. Non-targeting control crRNAs, *SLC2A1* crRNAs, and tracrRNA were resuspended in 10 mM Tris–HCl pH 7.4 buffer at the desired concentrations and incubated on the orbital shaker for 70 min at room temperature. To prepare the screening plates, 2.5 μL of respective 10 μM crRNA was added into flat-bottom, black-walled 96-well μCLEAR microplates using a Bravo Automated Liquid Handling Platform (Agilent Technologies). Specifically, 2.5 μL of 10 μM non-targeting crRNAs or *SLC2A1*-targeting crRNAs were added into select wells. Other wells were left empty as untreated controls. 7.5 μL of 333 nM tracrRNA, with 100 μM tracrRNA diluted in Opti-MEM I reduced serum medium, was added into each remaining well thereafter. Each plate was barcoded in a reference to the original library plate. Prepared plates were sealed with adhesive aluminum seals and stored at − 80 °C.

GLUT1^high^-C6-Caco-2 cells were cultured in T175 flasks until 100% confluent. To initiate the screen, stamped plates were pre-warmed at room temperature until reagents were thawed in each well. After brief centrifugation, 10 μL of Opti-MEM I reduced medium containing 0.5 μL of DharmaFECT1 reagent was added into each well of barcoded plates using a Multidrop Combi Reagent Dispenser. Transfection reagents were incubated for a minimum of 20 min at room temperature. While transfection mix was incubating, cells cultured in T175 flasks were washed once with DPBS, dissociated with TrypLE Express for 7–10 min, collected via centrifugation, and suspended into complete growth medium at the density of 5.5 × 10^5^ cells/mL. 80 μL of cell suspension was then added into each well of the stamped plates using the Multidrop Combi Reagent Dispenser to bring the final volume to 100 μL and the final concentration of crRNA:tracrRNA complex to 25 nM. 24 h after initiation of transfection, 100 μL of 2× anti-anti medium was added into each well using a EL406 Washer Dispenser (BioTek). 24 h later, medium was replaced with 200 μL 1× anti-anti medium using a EL406 Washer Dispenser. 48 h later, cells were fixed and stained as follows.

Before fixation, 8% formaldehyde solution was prepared by diluting 37% formaldehyde solution (Sigma-Aldrich #F1635) in UltraPure DNase/RNase-free distilled water. To limit cell detachment, 100 μL of DPBS was first added into each well to bring the total volume to 300 μL. 200 μL was then removed from each well. 100 μL of 8% formaldehyde was then added into each well yielding a final formaldehyde concentration of 4%, and plates were fixed at room temperature for at least 20 min. Once fixation was finished, liquid was fully aspirated and replaced with 200 μL of DPBS. All fixation procedures were completed with the EL406 Washer Dispenser.

For immunostaining, 200 μL of DPBS was fully aspirated and replaced with 50 μL of PBSDT using the EL406 plate washer. After 1 h, 50 μL of PBSDT was removed from each well using the EL406 plate washer, and 50 μL of Alexa Fluro 488-conjugated GLUT1 antibody diluted in PBSDT was added into each well using an electronic multichannel pipette. Fixed cells were incubated with conjugated antibody overnight at 4 °C. The following day, 50 μL of DAPI diluted in PBST was added to each well using an electronic multichannel pipette, and plates were incubated at room temperature for 20 min. 200 μL of PBST was then added to each well, and 300 μL of liquid was fully aspirated and replaced with 100 μL of DPBS using an EL406 plate washer.

Immunostained plates were imaged using an ImageXpress Micro XL automated imager (Molecular Devices). To fully automate the imaging process, plates were shuttled from a Matrix Platemate (Thermo Fisher Scientific) to the microscope stage using a Catalyst Express robotic arm (Thermo Fisher Scientific). Once each plate was loaded, six images were acquired from the same areas within each well with a 20× objective. All acquired images were barcoded in reference to initial library plates and saved as a tagged image file format for further analysis.

To score the fluorescence intensity on a per cell basis, a customized multi-wavelength cell scoring application module was built in MetaXpress software (Molecular Devices). For the configuration settings, wavelengths for DAPI and Alexa Fluro 488 were selected. Stained areas, approximate minimum width, approximate maximum width, and positive scoring criteria were specified to detect the signal of interest within images. Detection sensitivity was adjusted for each wavelength to specify the intensity above the local background. Following image quantification, selected measurements were exported and analyzed using a custom Python script (Supplementary Figs. 2 and 3). In every single plate, well values were generated by averaging the measurements of six images collected from each well and matching with gene symbols of the original library plate. Values of non-targeting control, positive control, untreated control, and unstained control were generated by averaging the measurements of four wells for each condition. Averaged well values aligned with gene symbols were then normalized to the non-targeting control within the same plate. Positive control, untreated control, and unstained control were normalized to non-targeting control as a quality control metric. Hits resulting in upregulation or downregulation of GLUT1 expression were selected based on the desired threshold as defined in the “Results” section and subjected to an Over Representation Analysis on the Reactome Pathway using the WEB-based Gene SeT AnaLysis Toolkit (WebGestalt)^37^.

### qPCR analysis

Cells were washed once with DPBS, lifted using a cell lifter, and collected via centrifugation. Resultant cell pellets were lysed in TRIzol reagent (Thermo Fisher Scientific #15596026) at room temperature for 10 min and stored at − 80 °C. Following the manufacturer’s guidelines, RNA from each sample was purified using a Direct-zol RNA Miniprep kit (Zymo Research #R2051). cDNA was generated using a High-Capacity cDNA Reverse Transcription kit (Applied Biosystems #4368814). qPCR was performed in a 20 µL of reaction mix containing 9 µL of cDNA template, 10 µL of TaqMan Universal PCR Master Mix (Applied Biosystems #4304437), and 1 µL of TaqMan probe (Supplementary Table 4) on a CFX96 Thermocycler (Bio-Rad). Relative quantification was performed using the ∆∆Cq method.

### Small molecule treatments in Caco-2 cells

GLUT1^high^-C6-Caco-2 cells were cultured in 150 mm dishes until 100% confluent. Cells were washed with DPBS, dissociated with TrypLE Express for 5–10 min and resuspended in complete growth media at a density of 4.4 × 10^5^ cells/mL. 100 µL of cell suspension was dispensed into glass bottom 96 well plates. Following a 24-h recovery period, cells were treated with either 30 µM oxymetazoline (Cayman Chemicals), 30 µM phenoxybenzamine (Cayman Chemicals), or an equivalent volume of DMSO. 48 h post treatment, cells were fixed and immunostained as previously described. Immunostained plates were imaged using an ImageXpress Micro XL automated imager. Fluorescence intensity was scored on a per cell basis using MetaXpress software as previously described.

### Statistical analysis

All data are presented as mean ± standard deviation. One-way ANOVA and the Student’s unpaired t-test were used to determine statistical significance as described in each figure.

## Results

### Establishment of a Cas9-expressing Caco-2 line with homogeneous GLUT1 expression

**Figure 1:**
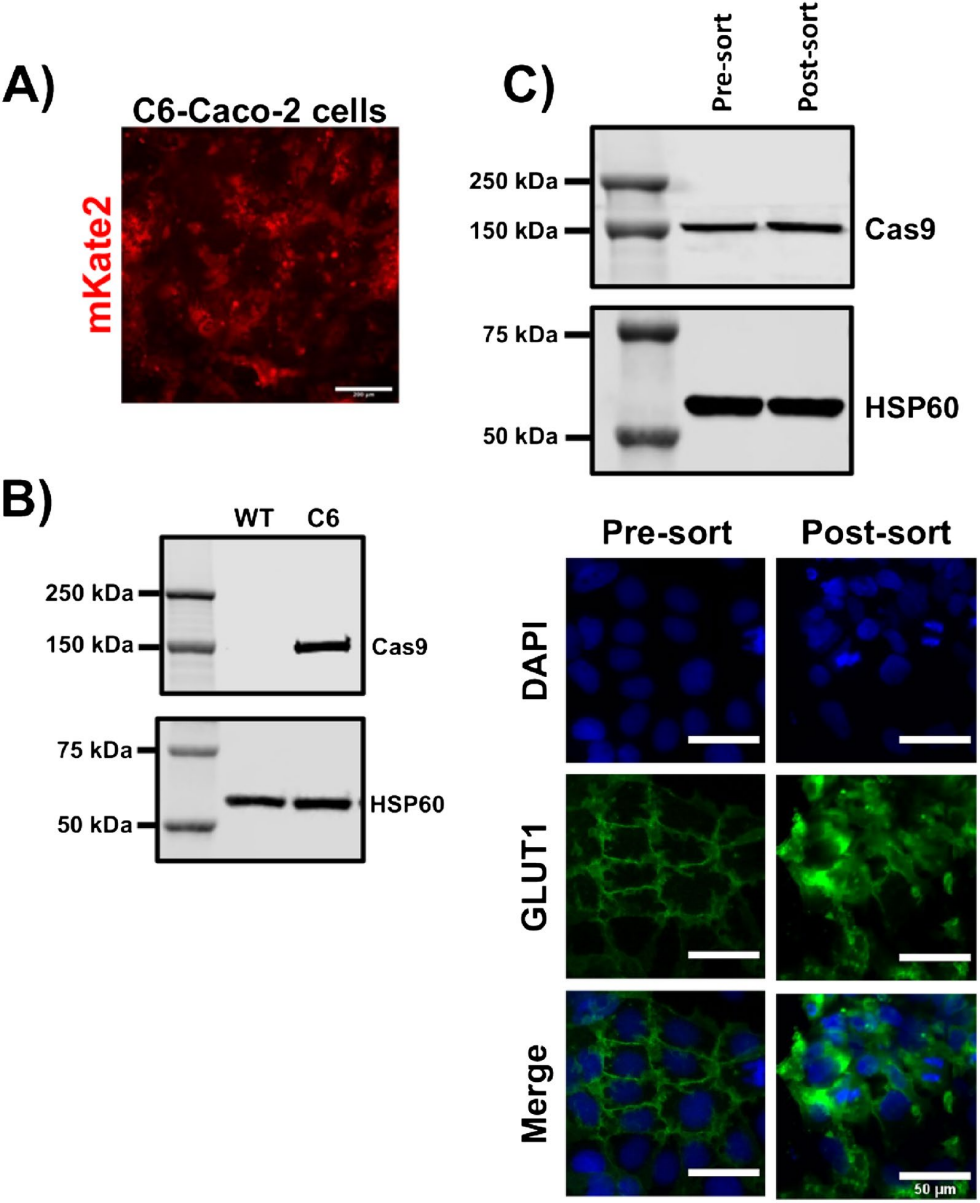
Establishment of a Cas9-expressing Caco-2 cell line with homogeneous GLUT1 expression. (**A**) Representative immunofluorescent image of mKate2 expression in clone C6 after sorting and outgrowth. Scale bar represented 200 µm. (**B**) Representative western blot assessment of Cas9 protein expression in wild-type Caco-2 cells versus clone C6. HSP60 was used as a loading control. Original blots are presented in Supplementary Fig. 5. (**C**) Assessment of Cas9 and GLUT1 expression after bulk sorting of C6-Caco-2 cells using an anti-GLUT1 antibody. Cas9 expression was assessed by western blot with HSP60 as a loading control. Original blots are presented in Supplementary Fig. 6. GLUT1 expression was assessed by immunostaining. Scale bars represent 50 µm.

Arrayed CRISPR screens should ideally be performed with adherent cells that are easily scaled, amenable to genetic modification, and possess the phenotype of interest. We queried the Human Protein Atlas cell line database, which contains genome-wide RNA expression profiles of human protein-encoding genes in 69 human cell lines. From this information, we selected the Caco-2 cells based on high expression of *SLC2A1* (encoding GLUT1). We have previously shown that these cells can be genetically modified using CRISPR strategies^36^, further supporting their use. To enable a forward genetic screen solely with transfected crRNA, we transduced Caco-2 cells with lentiviral particles to promote constitutive Cas9 expression. Single Cas9-expressing clones were sorted based on expression of mKate2 reporter (Fig. 1A), and Clone C6 was selected for further use based on robust Cas9 expression as determined by western blot (Fig. 1B). Since we have also shown that GLUT1 expression in Caco-2 cells can be heterogeneous^36^, we additionally performed a bulk sort based on GLUT1 expression to increase homogeneity, and we confirmed maintenance of both GLUT1 and Cas9 expression after several passages (Fig. 1C). This approach generated Caco-2 cells with uniform GLUT1 expression (GLUT1^high^-C6-Caco-2) that were suitable for CRISPR knockout screening.

### Assessment of knockout efficacy and quantitative imaging parameters

**Figure 2:**
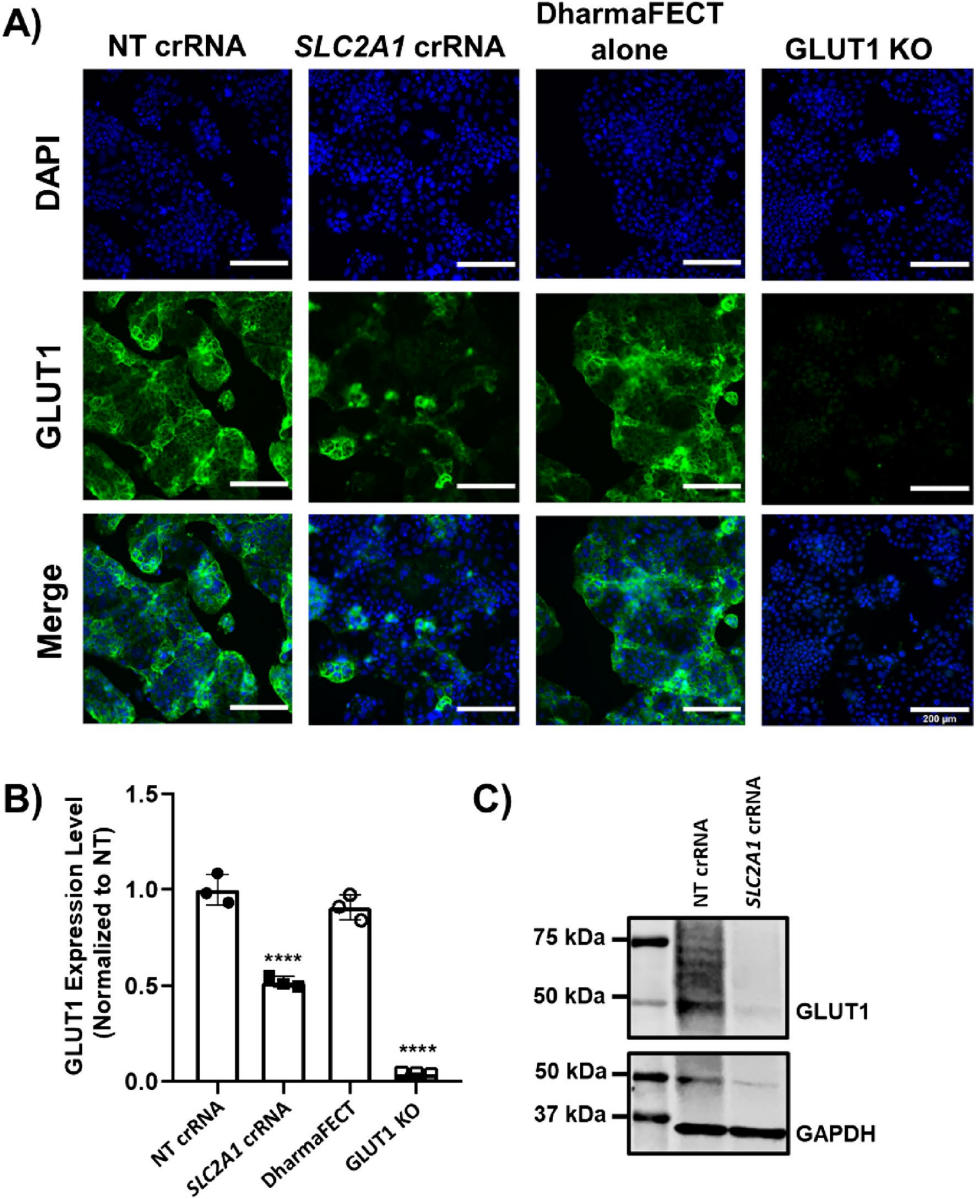
Validation of GLUT1 immunostaining as a quantitative metric for high content image-based CRISPR screening. (**A**) Representative images of GLUT1^high^-C6-Caco-2 cells under various conditions. NT denotes non-targeting control. DharmaFECT alone denotes the transfection reagent control. GLUT1 KO denotes cells lacking endogenous GLUT1 expression. Scale bars indicate 200 μm. (**B**) Quantification of fluorescence intensity for each condition. Each data point is the mean of a single biological replicate calculated using six images taken from each well (n = 6 technical triplicates). Data are represented as mean ± standard deviation from n = 3 biological replicates per experimental condition. A one-way ANOVA with a Dunnett’s multiple comparisons test was used to evaluate the statistical significance across conditions (****, p < 0.0001). (**C**) Representative western blot of GLUT1 expression in GLUT1^high^-C6-Caco-2 cells transfected with non-targeting (NT) or *SLC2A1*-targeting crRNA. GAPDH served as the loading control. Original blots are presented in Supplementary Fig. 7.

To assess screening feasibility, GLUT1^high^-C6-Caco-2 cells were transfected with crRNA:tracrRNA under the conditions to be utilized in the screen, and images were acquired with an automated fluorescence microscope (Fig. 2A). We analyzed cells receiving the non-targeting crRNA, the *SLC2A1*-targeting crRNA, or transfection reagent alone; we also utilized a GLUT1 knockout Caco-2 line from our prior work^36^ as an additional control for background signal threshold. GLUT1 expression levels were calculated on a per cell basis after normalizing integrated fluorescence intensity to cell density (i.e. number of nuclei) within each acquired image. Compared to the non-targeting crRNA condition, cells treated with *SLC2A1*-targeting crRNA exhibited a statistically significant 48% reduction in GLUT1 expression, and there was no significant difference between the non-targeting crRNA and the transfection only control (Fig. 2B). For comparison, the GLUT1 KO cells were quantified at 4% GLUT1 expression relative to the non-targeting control (Fig. 2B). GLUT1 reduction from the *SLC2A1*-targeting crRNA was confirmed with western blot (Fig. 2C). This outcome, coupled with the low variance in measured GLUT1 expression within each condition, provided confidence that we would be able to detect relevant differences in GLUT1 expression in an arrayed knockout screen.

### Arrayed CRISPR screen identifies genes that putatively regulate GLUT1 expression

**Figure 3:**
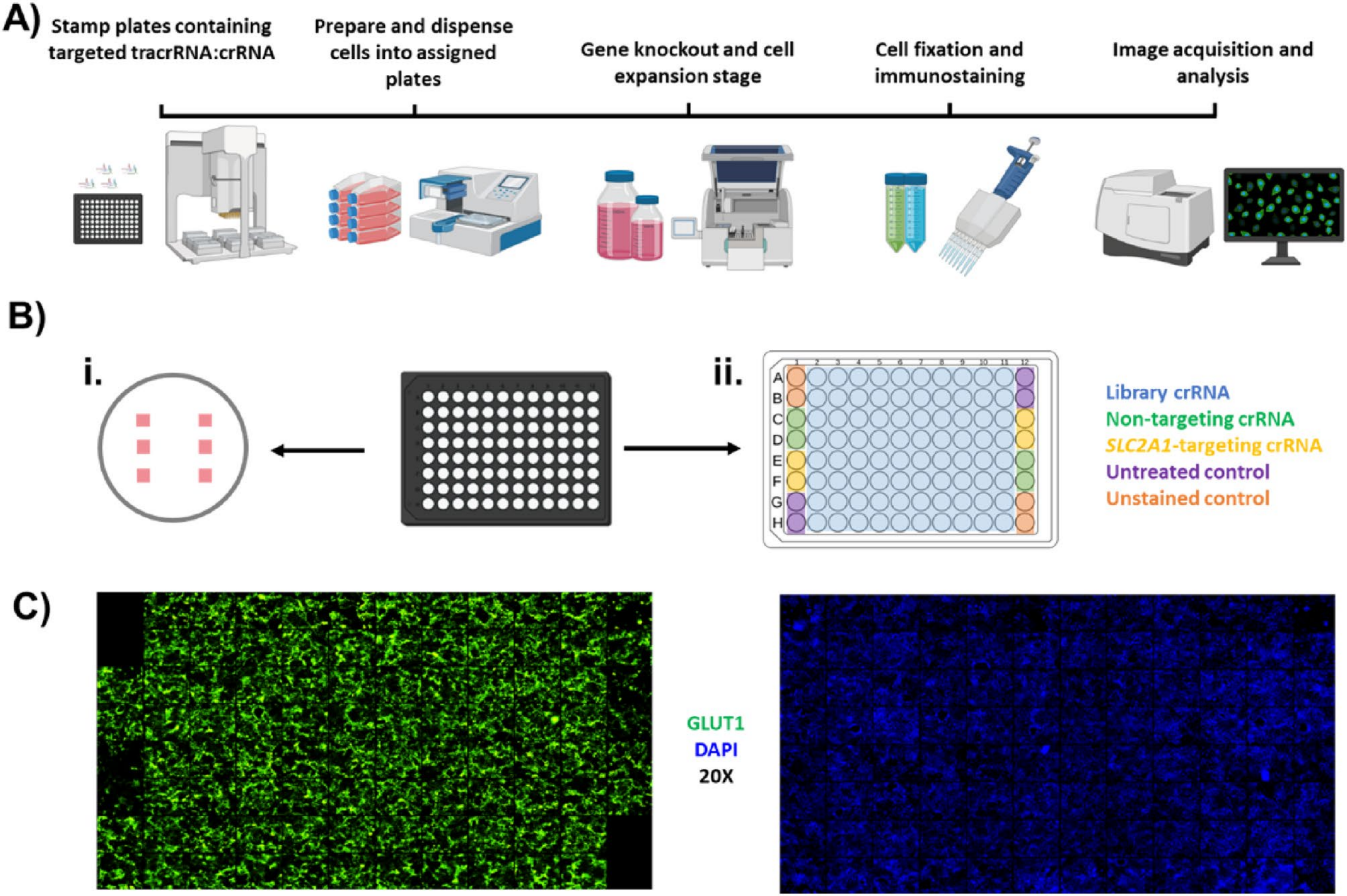
Arrayed screen workflow. (**A**) Schematic of screening procedure. Library plates were stamped and barcoded with a Bravo automated liquid handling platform, cells were distributed with a Multidrop Combi reagent dispenser for reverse transfection, medium was exchanged with an EL406 washer dispenser, and cells were fixed, stained, and imaged with an ImageXpress Micro XL automated imager. (**B**) Schematic layout for imaging in 96-well plates. Six images were taken from each well at the indicated locations (panel i) and control and experimental crRNAs were distributed within each plate as presented (panel ii). (**C**) Representative images taken from one 96-well plate.

As described in the “Methods” section, we developed and implemented an automated liquid handing and high-content imaging screen (Fig. 3A). We screened more than 8,000 genes in duplicate with synthetic crRNAs spanning the following areas: phosphatases, GRCRs, ion channels, proteases, kinases, ubiquitin enzymes, transcriptional factors, and human drug targets. We used an experimental design where each 96-well plate contained four wells receiving crRNA targeting *SLC2A1*, four wells receiving the non-targeting crRNA control, four wells treated with transfection reagent but no crRNA, four wells that were untreated and unstained to control for background signal, and 80 wells receiving the crRNA library (Fig. 3B). Within each well, six images were acquired to limit variability due to cell density or heterogenous crRNA distribution. Representative images are shown from one 96-well plate with a good alignment, where there was no signal from unstained wells and an evident decrease in fluorescence intensity in wells with *SLC2A1*-targeting crRNA (Fig. 3C).

**Figure 4:**
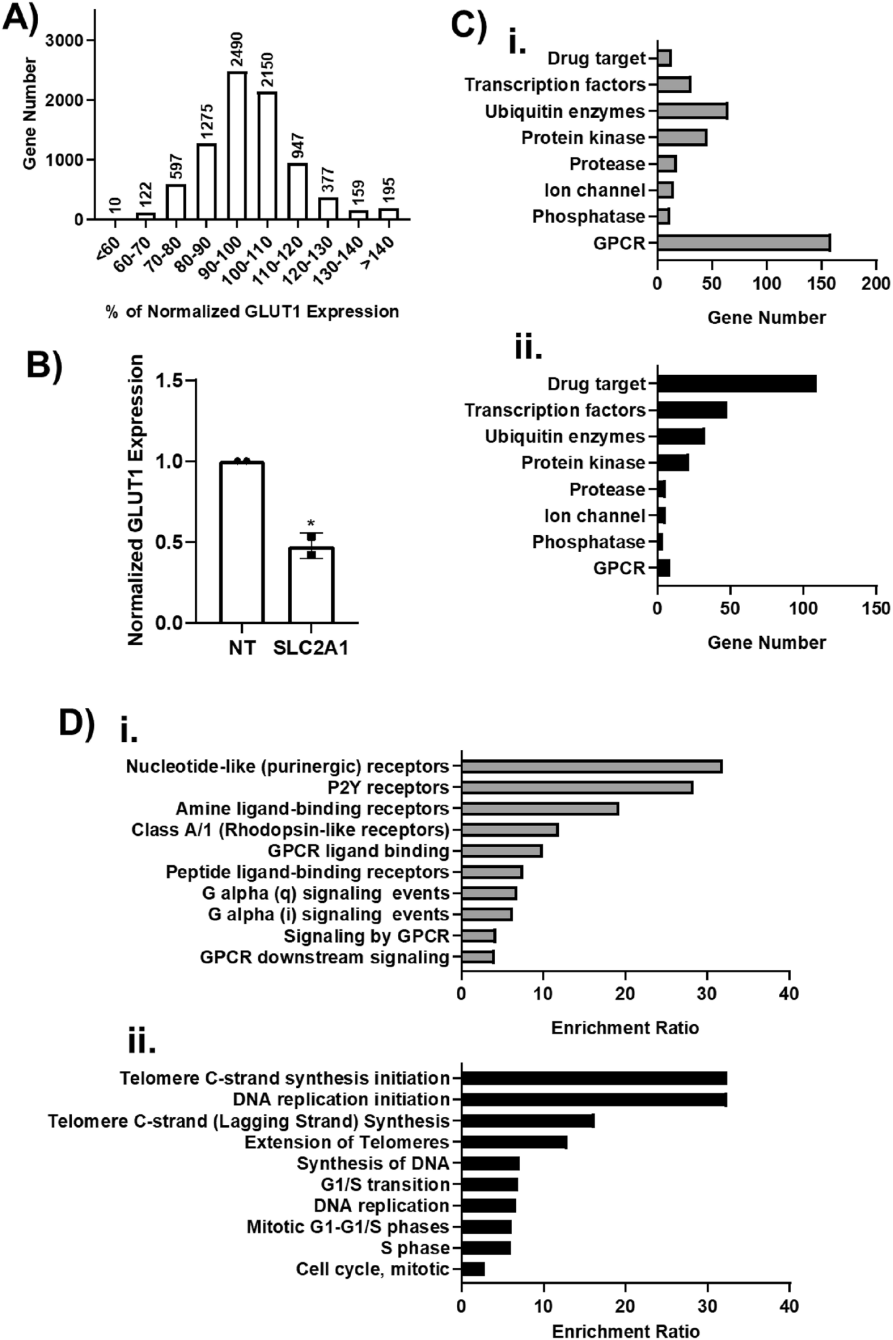
Primary screen outcomes. (**A**) Compiled CRISPR screening results for 8322 genes. Data represent the mean from n = 2 biological replicates per experimental condition and are binned by GLUT1 expression normalized to the non-targeting control. (**B**) Normalized GLUT1 expression from the library wells containing *SLC2A1*-targeting crRNA. Each data point is the mean of a single biological replicate calculated using six images taken from each well (n = 6 technical triplicates). Data are presented as mean ± standard deviation from the 2 biological replicates. Statistical significance was calculated using the Student’s unpaired t-test (*, p < 0.05). NT denotes non-targeting control. (**C**) Compiled datasets of hit distribution corresponding to biological function. Top panel (i) is hits that downregulated GLUT1 and bottom panel (ii) is hits that upregulated GLUT1. (**D**) Signaling pathways enriched for genes whose removal downregulated (i) or upregulated (ii) GLUT1 expression.

Figure 4A shows the overall distribution of normalized GLUT1 expression. Data are presented as the average of the two replicates per gene, and the normalized expression values for each replicate, as well as the average and standard deviation for each gene, can be found in Supplementary Table 5. Of note, *SLC2A1* was present in the library, and each of the replicate wells (which were screened on different plates on different days) produced a decrease in normalized GLUT1 expression of ~ 50% (Fig. 4B), which was consistent with our initial tests in Fig. 2C and again provides confidence in the screen outcomes. Overall, the standard deviation of normalized GLUT1 expression for the entire screen was 17%. Based on this value, we designated each gene as a hit if both replicates yielded a normalized GLUT1 expression value above 117% or below 83%, which accounts for the consistency of the measurement. Using this hit threshold, we identified 359 genes whose removal decreased GLUT1 expression and 237 genes whose removal increased GLUT1 expression (Supplementary Table 5). Genes responsible for GLUT1 downregulation were enriched for GPCR signaling whereas genes responsible for GLUT1 upregulation were enriched in the drug target library (Fig. 4C). These hits were further subject to over-representation analysis for enriched Reactome signaling pathways (false-discovery rate less than 0.05). Genes whose removal downregulated GLUT1 expression were enriched along the signaling pathway of nucleotide-like (purinergic), P2Y, amine ligand-binding, and Rhodopsin-like receptors (Fig. 4D), which are all GPCRs activated by small ligands and various cytokines. Genes whose removal upregulated GLUT1 expression were enriched in signaling pathways primarily related to DNA replication (Fig. 4D).

### Secondary hit validation in in vitro models

**Figure 5:**
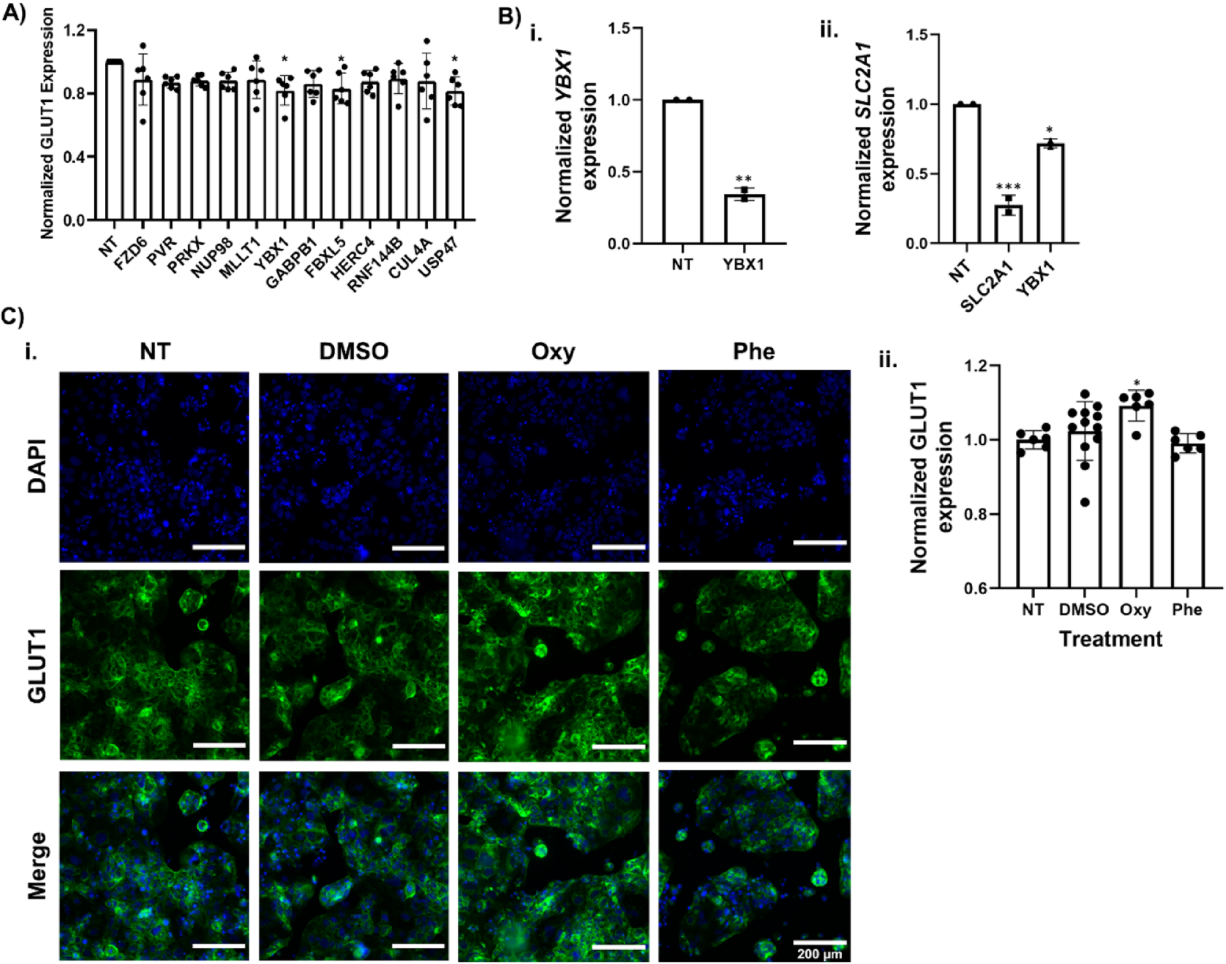
Secondary hit validation. (**A**) Compiled datasets represent GLUT1 expression levels in GLUT1^high^-C6-Caco-2 cells corresponding to different gRNA-targeted genes. Each data point is the mean of a single biological replicate calculated from triplicate wells (18 quantified images). Data are represented as mean ± standard deviation from n = 3 biological replicates per targeted gene. A one-way ANOVA with a post Dunnett test was used to evaluate the statistical significance across conditions (*, p < 0.05). All data were normalized to the non-targeting control (NT). (**B**) Panel i shows *YBX1* expression in GLUT1^high^-C6-Caco-2 cells that received non-targeting gRNAs or gRNAs targeting *YBX1*. Data are from qPCR measurements and are represented as mean ± standard deviation from 2 biological replicates. Statistical significance was calculated using the Student’s unpaired t-test. (**, p < 0.01). Panel ii shows *SLC2A1* expression in GLUT1^high^-C6-Caco-2 cells that received non-targeting gRNAs or gRNAs targeting *SLC2A1* or *YBX1*. Data are represented as mean ± standard deviation from 2 biological replicates. A one-way ANOVA with a Dunnett’s multiple comparisons test was used to evaluate the statistical significance across conditions (***, p < 0.001; *, p < 0.05). All data were normalized to the non-targeting control (NT). (**C**) Panel i shows representative immunofluorescence images of GLUT1 expression following treatment with DMSO (vehicle control), oxymetazoline (Oxy, adrenergic agonist), and phenoxybenzamine (Phe, antagonist), or the untreated control (NT). Scale bars represent 200 µm. Panel ii shows the quantified GLUT1 expression from these images. Data represented as mean ± standard deviation from at least 6 biological replicates (at least 36 quantified images). Statistical significance was calculated using a one-way ANOVA with Dunnett’s multiple comparisons test (*, p < 0.05).

To build on outcomes from the primary screen, we randomly selected 12 gene candidates to validate in the GLUT1^high^-C6-Caco-2 cells across multiple biological replicates using the same gRNA delivery strategy as the primary screen (Fig. 5A). Of these, gRNAs targeting *YBX1*, *FBXL5*, and *USP47* yielded significant downregulation of GLUT1 protein. Of note, YBX1 has already been implicated in GLUT1 regulation in bladder cancer cells, where shRNA-mediated knockdown of *YBX1* significantly reduced *SLC2A1* expression across two different cell lines^38^. To confirm a similar outcome in Caco-2 cells, we repeated the transfection with *YBX1-*specific gRNAs and measured *SLC2A1* expression. As expected, treatment with these *YBX1-*specific gRNAs resulted in a ~ 60% reduction in *YBX1* expression and a ~ 30% decrease in *SLC2A1* expression (Fig. 5B). For comparison, treatment with *SLC2A1*-specific gRNAs under the same conditions yielded a ~ 75% reduction in *SLC2A1* expression (Fig. 5B).

Last, given the enrichment in GPCR hits in the screen, we sought to test pharmacological manipulation of GLUT1 expression. Here, we focused on the adrenergic receptor family, since knockout of several of these receptors significantly modulated GLUT1 expression in the GLUT1^high^-C6-Caco-2 system. Of note, adrenergic signaling plays a crucial role in regulating cardiac function both under normal conditions and in case of pathological events^39^, and α-adrenergic receptor signaling has been shown to influence glucose transport within cardiomyocytes, which is a vital mechanism in the prevention and response to ischemic injury to the heart^40–42^. Taking this into account, we tested whether pharmacological modulation of adrenergic receptor signaling could alter GLUT1 expression. First, we confirmed expression of *ADRA1* in GLUT1^high^-C6-Caco-2 cells using PCR and gel electrophoresis (Supplementary Fig. 4). We then treated GLUT1^high^-C6-Caco-2 cells with oxymetazoline (an adrenoreceptor agonist), phenoxybenzamine (an adrenoreceptor antagonist), or DMSO as a vehicle control and quantified GLUT1 protein expression. In response to oxymetazoline treatment, we observed an increase in GLUT1 protein expression as measured by immunofluorescence, whereas DMSO and phenoxybenzamine treatment resulted in no such changes to GLUT1 protein expression (Fig. 5C). Hence, GLUT1 expression can be modulated by pharmacological targeting of adrenergic signaling in vitro, providing further confirmation of the screen outcomes.

## Discussion

GLUT1 is an essential membrane transporter that facilitates cellular glucose uptake, especially in tissues that rely solely on glucose as their source of energy. Approaches to modulate GLUT1 expression as a means of alleviating pathology have been studied with great interest^43–45^, necessitating a more comprehensive understanding of the specific mechanisms and intracellular machinery that regulate GLUT1 expression. To address this challenge in an unbiased manner, we performed an arrayed CRISPR knockout screen to assess how loss of individual genes directly influenced GLUT1 expression. We sought to identify genes that negatively regulate GLUT1 in order to gain insights into the genetic regulators that influence GLUT1 pathologies. This approach identified more than 300 genes whose removal reduced GLUT1 expression.

Interestingly, the enriched genes were predominantly stratified along signaling pathways activated by GPCRs, particularly from the rhodopsin-like receptor family. This class of GPCRs is clustered by phylogenetic analyses, and its ligands include cytokines, hormones, neurotransmitters, and metabolites^46^, suggesting diverse signaling pathways can regulate GLUT1 expression. Although this screen was conducted using immortalized Caco-2 cells, the primary goal of this study was to identify genetic regulators of GLUT1 irrespective of the cell type of origin. We believe that our work is agnostic to specific organ systems and these results can form the framework for understanding GLUT1 regulation across different cell types. For example, Caco-2 cells have been used as a BBB model, specifically in the context of BBB permeability and transporter function^47, 48^. GLUT1 expression at the BBB is decreased in AD patients^22, 28–30^. AD and related dementias afflict millions of people and are expected to increase concurrently with life expectancies worldwide. The contribution of vascular dysfunction to AD onset and progression has garnered significant interest, and the correlation between cerebrovascular dysfunction and severity of AD and cognitive impairment has been suggested in patients^49–56^. Many of the GPCRs identified in our screen are predicted to be expressed on brain endothelial cells according to scRNA-seq databases^57^. For example, the P2RY2 purinergic receptor was identified as a hit and is predicted to be expressed at moderate levels in mouse brain endothelial cells. P2Y receptors are activated by purines and pyrimidines depending on the receptor species^58^. Purinergic signaling has long been recognized for its role in neurovascular coupling and cerebral blood flow^59^, and mechanisms underlying crosstalk between endothelial cells and various neurons and glia are still being identified as exemplified by recent studies showing novel associations between capillaries and other components of the neurovascular unit where purinergic signaling is relevant^60^. Future studies can focus on determining if P2Y-dependent signaling pathways also influence GLUT1 expression and glucose metabolism. In general, the use of genetic knockdown or pharmacological modulation of targets in more complex models, such as ex vivo slice cultures of mouse brain that more closely mimic physiological conditions, will provide more translational relevance of this work towards BBB function and disease-related pathologies.

We further note many GPCRs identified as hits in our screen that are activated by small molecules and metabolites including but not limited to catecholamines, acetylcholine, dopamine, adenosine, prostaglandins, sphingosines, and fatty acids. The identification of neurotransmitter receptors implies that neural activity could in part control glucose uptake to meet energy demands. This notion agrees with a recent study demonstrating that glutamatergic neuron activity could regulate BBB transporter expression levels, one of which was *SLC2A1* (upregulated when glutamatergic activity was chemogenetically silenced)^61^. The identification of catecholamine and dopamine receptors also implies that emotional state can regulate GLUT1 expression. Adrenergic receptor stimulation in other organs can regulate GLUT1 trafficking^40^, and similar concepts could apply at the BBB. The identification of adenosine receptors is perhaps unsurprising, since prior work in cultured retinal endothelial cells has shown that activation of adenosine receptors can alter *SLC2A1* expression^62^. The identification of lipid-based molecules and metabolites is also quite interesting in the context of AD risk. Numerous studies have sought to identify features of the brain and serum metabolome that may contribute to AD development and progression. For example, metabolomics studies have identified certain lipids as markers of prodromal and preclinical AD, as well as predictors of future conversion to incident AD in cognitively normal individuals^63–66^; more recent studies have shown similar results. Since these changes in serum and brain metabolome are associated with future conversion to AD, and brain hypometabolism is also an early event in AD, this raises an intriguing possibility that brain endothelial cells sense these changes and downregulate GLUT1 in response, thereby contributing to AD progression. Future work will be necessary to establish these connections explicitly.

Additionally, this work may have implications for other neural and peripheral diseases. Similar to AD, other neurodegenerative diseases such as Parkinson’s are characterized by dysregulated glucose metabolism^67^ and serum metabolome signatures^68^—these could potentially be linked to altered GLUT1 expression. Outside the brain, GLUT1 is critical for T cell activation^69^ and plays important roles in how subsets of T cells regulate immunologic diseases^70^. GLUT1 overexpression is a hallmark of many cancers and may contribute to growth and resistance to treatment^33, 34, 71^, while GLUT1 expression in tumor-associated cells such as neutrophils can influence pro- versus anti-tumor behaviors^32^. Hence, the data generated from our CRISPR screen may be of interest across many different biological systems where regulation of GLUT1 expression is relevant.

Last, in addition to identifying several genes that can impact GLUT1 expression, this work also serves as a technical resource for setting up future high-throughput screens. Establishing high-throughput screening protocols is labor-, cost- and equipment-intensive. We have provided a step-by-step illustration of the technical considerations that went into validating and conducting this screen including crucial technical and biological considerations such as selection of an appropriate cell line, choice of quantifiable phenotypes and development of automated workflows for data analysis. These advancements may prove generally useful to other image-based screens examining transporter expression and function.

### Supplementary Information

Supplementary Information 1.

Supplementary Table 5.

## Data Availability

The datasets used and/or analyzed during the current study are available from the corresponding author upon reasonable request.
