# Supplementary material for: An arrayed CRISPR knockout screen identifies genetic regulators of GLUT1 expression
Source: Sci Rep. 2023 Nov 29;13:21038. doi: 10.1038/s41598-023-48361-5 (PMC10687026; doi:10.1038/s41598-023-48361-5)
Supplement: Supplementary file 1 — Supplementary Information 1. [file 41598_2023_48361_MOESM1_ESM.docx]

**Supplementary Information**

**An arrayed CRISPR knockout screen identifies genetic regulators of GLUT1 expression**

Yajuan Shi, Ketaki A. Katdare, Hyosung Kim, Jonah C. Rosch, Emma H. Neal, Sidney Vafaie-Partin, Joshua A. Bauer, and Ethan S. Lippmann

**Supplementary Table 1.** **Primary antibodies.**

| **Antigen** | **Species** | **Vendor** | **Product number** | **Application** | **Dilution** |
| --- | --- | --- | --- | --- | --- |
| GLUT-1 | Mouse | R&D Systems | FAB1418G | Western blot | 1:1000 |
| GLUT-1 | Rabbit | Abcam | 15309 | Western blot | 1:1000 |
| GLUT-1 | Mouse | Abcam | Ab195359 | Immunostaining | 1:100 |
| GAPDH | Mouse | Cell Signaling Technology | D4C6R | Western blot | 1:1000 |
| HSP60 | Rabbit | Cell Signaling Technology | D6F1 | Western blot | 1:1000 |
| Cas9 | Mouse | Diagenode | C15200203 | Western blot | 1:1000 |

**Supplementary Table 2.** **Secondary antibodies.**

| **Species reactivity** | **Host** | **Conjugate** | **Vendor** | **Dilution** |
| --- | --- | --- | --- | --- |
| Mouse | Donkey | Alexa Fluor 488 | Thermo Fisher Scientific | 1:200 |
| Rabbit | Donkey | Alexa Fluor 488 | Thermo Fisher Scientific | 1:200 |
| Goat | Donkey | Alexa Fluor 488 | Thermo Fisher Scientific | 1:200 |
| Goat | Donkey | Texas Red | Thermo Fisher Scientific | 1:200 |
| Chicken | Goat | Alexa Fluor 647 | Thermo Fisher Scientific | 1:1000 |
| Rabbit | Goat | IRDye 800CW | LI-COR | 1:15,000 |
| Mouse | Goat | IRDye 800CW | LI-COR | 1:15,000 |

**Supplementary Table 3. crRNA identifiers.**

| **Target** | **Identifier** |
| --- | --- |
| NT control | U-007501/2/3/4 |
| *SLC2A1* | CM-007509 |
| *FZD6* | F-005505-00 |
| *PVR* | F-015323-00 |
| *PRKX* | F-004660-00 |
| *NUP98* | F-013078-00 |
| *MLLT1* | F-016352-00 |
| *YBX1* | F-010213-00 |
| *GABPB1* | F-013083-00 |
| *FBXL5* | F-012424-00 |
| *HERC4* | F-021426-00 |
| *RNF144B* | F-025119-00 |
| *CUL4A* | F-012610-00 |
| *USP47* | F-006093-00 |

**Supplementary Table 4.** **TaqMan probes.**

| **Gene** | **Catalog number** |
| --- | --- |
| *SLC2A1* | Hs00892681_m1 |
| *YBX1* | Hs00358903_g1 |
| *ADRA1A1* | Hs05032266_s1 |
| *GAPDH* | Hs02786624_g1 |

**Supplementary Table 5. Full results from arrayed screen and filtered hits based on downregulation of GLUT1 expression.** Table is found in attached Excel file.

**Supplementary Figure 1: Flow cytometry gating strategy for generating GLUT1^high^-C6-Caco-2 cells.** Cells were first gated to filter out doublets and dead cells. Live single cells were assessed for GLUT1 expression as labelled by Alexa Fluor 488-conjugated anti-GLUT1 antibody and cells with highest expression (top 2%) were collected in bulk.

**Supplementary Figure 2:** **Python code used to normalize measurements of per-cell GLUT1 expression in control wells to non-targeting control wells on the same plate.**

**Supplementary Figure 3: Python code used to normalize measurements of per-cell GLUT1 expression in each experimental well to non-targeting control wells on the same plate.**

**
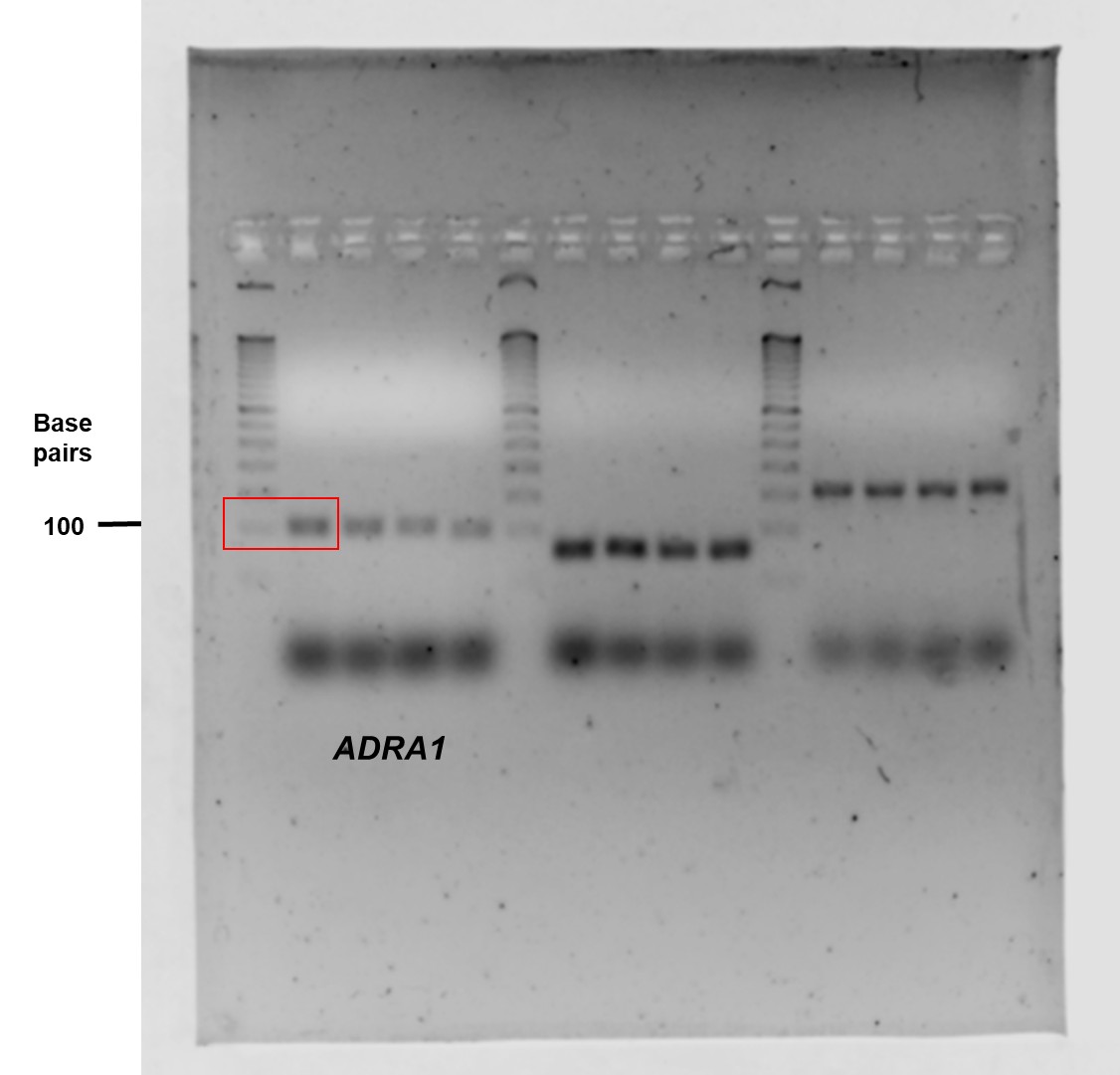
**

**Supplementary Figure 4: Gel electrophoresis shows *ADRA1* mRNA expression in GLUT1^high^-C6-Caco-2 cells.** Outlined band shows amplified *ADRA1* PCR product from GLUT1^high^-C6-Caco-2 cells. Predicted PCR band size for *ADRA1* mRNA is 100 base pairs.


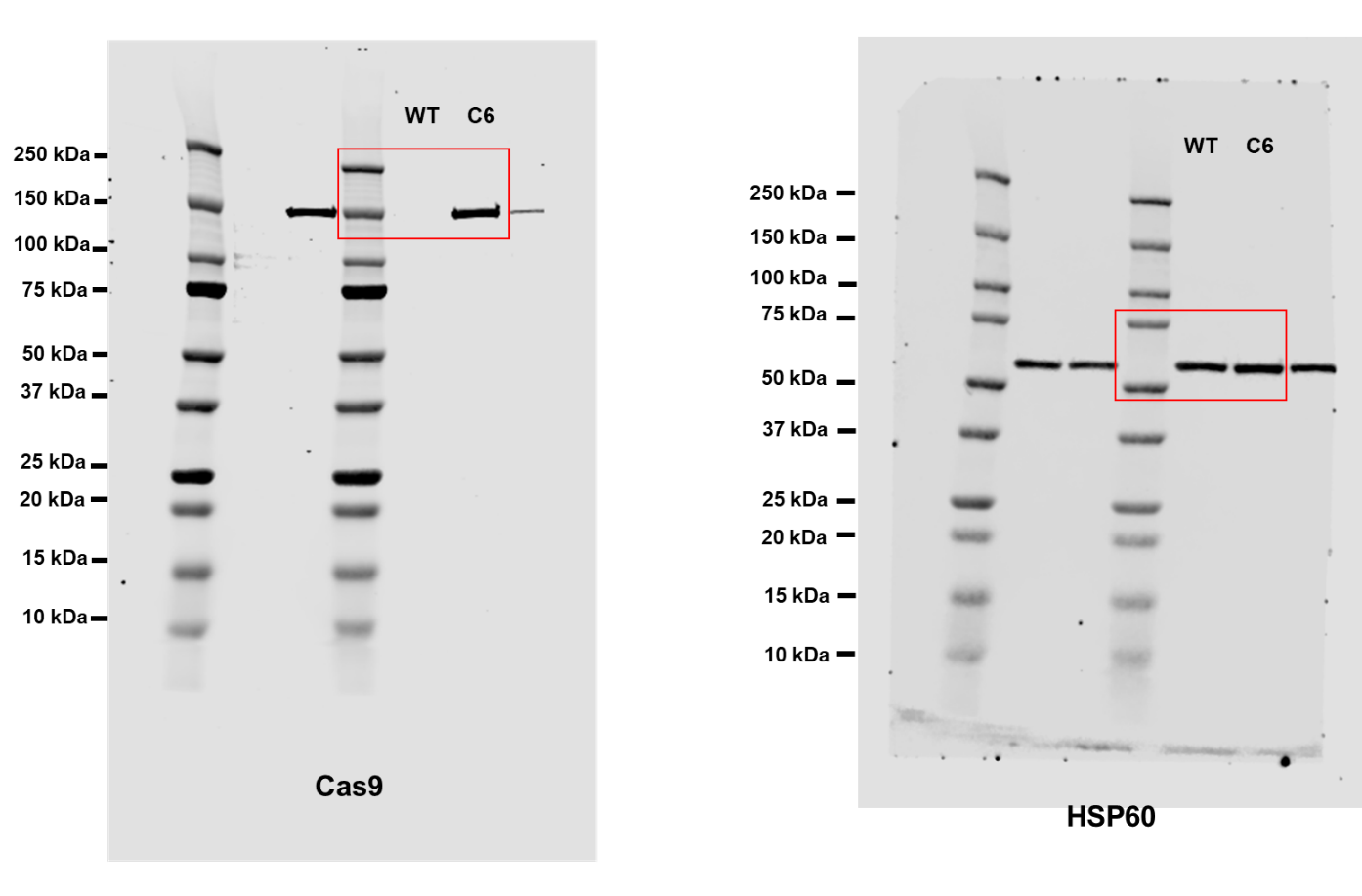


**Supplementary Figure 5: Original uncropped blots for Figure 1B. Cropped regions are outlined in red.**


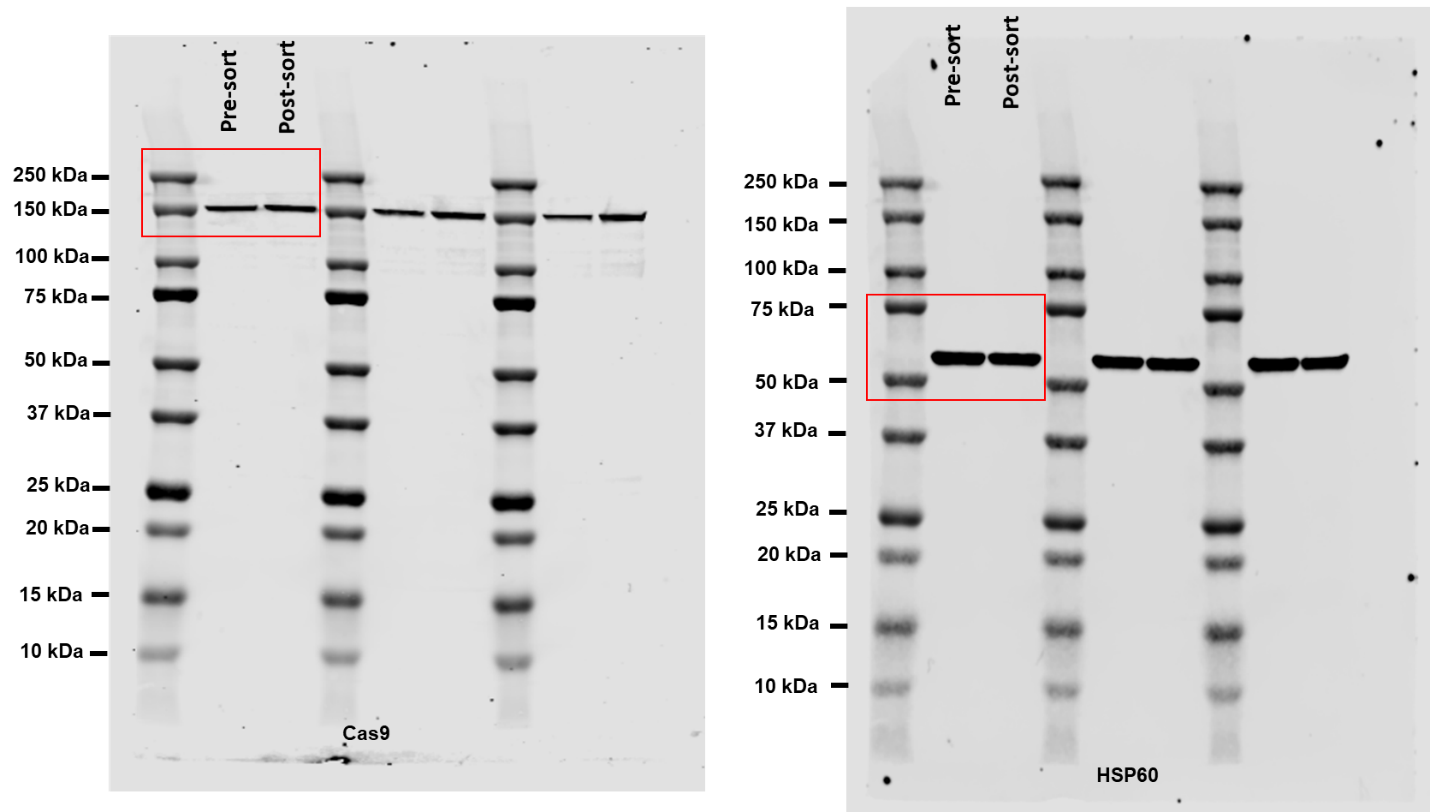


**Supplementary Figure 6: Original uncropped blots for Figure 1C. Cropped regions are outlined in red.**


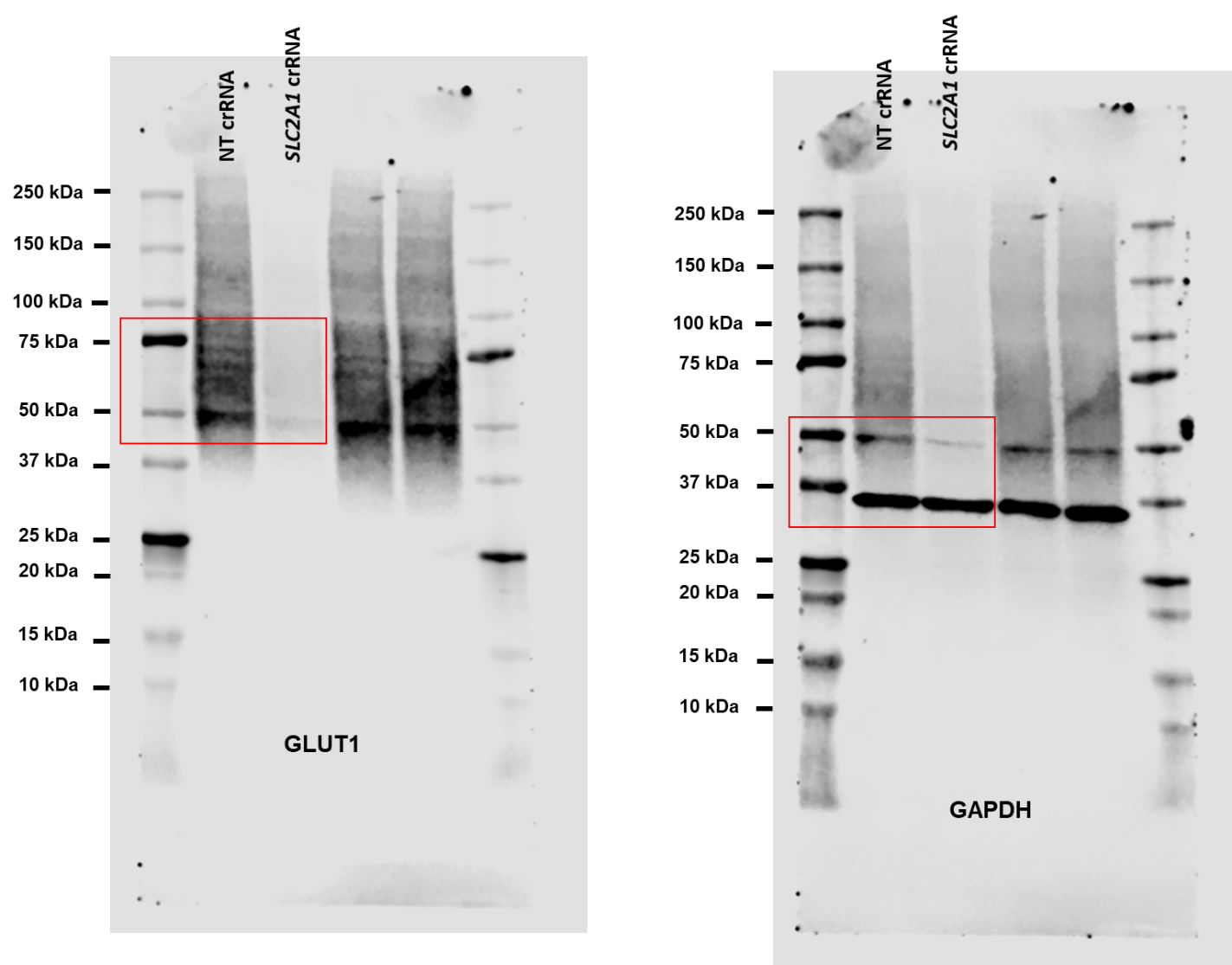


**Supplementary Figure 7: Original uncropped blots for Figure 2C. Cropped regions are outlined in red.**
